# Crystal structure at 100 K of bis­[1,2-bis­(di­phenyl­phosphan­yl)ethane]­nickel(II) bis­(tri­fluoro­methane­sulfonate): a possible negative thermal expansion mol­ecular material

**DOI:** 10.1107/S2056989018014846

**Published:** 2018-10-31

**Authors:** Cristian A. Cano-Benítez, Alejandro J. Metta-Magaña, Álvaro Duarte-Ruiz

**Affiliations:** a Departamento de Química, Universidad Nacional de Colombia, Ciudad Universitaria, Bogotá Kr 30 No 45-03, Colombia; b Department of Chemistry and Biochemistry, University of Texas at El Paso, 500 W University El Paso, Texas 79968, USA

**Keywords:** 100 K, RT, dppe, square-planar geometry, Negative thermal expansion, NTE, crystal structure

## Abstract

In the title salt, [Ni(C_26_H_24_P_2_)_2_](CF_3_SO_3_)_2_ or [Ni(dppe)_2_]^2+^·(OTf^−^)_2_ [dppe = 1,2-bis­(di­phenyl­phosphan­yl)ethane and OTf^−^ = tri­fluoro­methane­sulfonate], the Ni atom (site symmetry ) has a square-planar geometry with the bidentate ligands chelating the metal. As a result of the steric hindrance of the phenyl rings, the counter-ions are blocked from the metal coordination sphere. The dynamic disorder of the anion existing at 296 K is reduced at 100 K and based on these two temperatures, negative thermal expansion behaviour is observed.

## Abstract 2

Description and comparison of the crystal structure of (Ni(1,2–bis­(di­phenyl­phosphan­yl)ethane)_2_)(CF_3_SO_3_)_2_ at 100 K with its nitrate and bromide analogues.

## Chemical context   

The cation presented here has been synthesized with different counter-ions [Ni(C_26_H_24_P_2_)_2_]·*X*_2_ for different reasons: as by-product in a halogenation process (*X* = Cl^−^, Br^−^, I^−^) (Zarkesh *et al.*, 2014 ▸); to research its anti­cancer properties (*X* = Br^−^, I^−^, NO_3_^−^) (Jarrett & Sadler, 1991 ▸); as result of protonation studies (*X* = ClO_4_^−^) (Cariati *et al.*, 1966 ▸); and as byproducts while trying to increase the coordination number of [Ni(dppe)*X*_2_] (*X* = Cl^−^, Br^−^, I^−^; Hudson *et al.*, 1968 ▸). Moreover, to date there are just two reports of its crystal structure with NO_3_^−^ (VASCIB; Williams, 1989 ▸) and Br^−^ counter-ions (XUQYOZ; Higgs *et al.*, 2010 ▸).
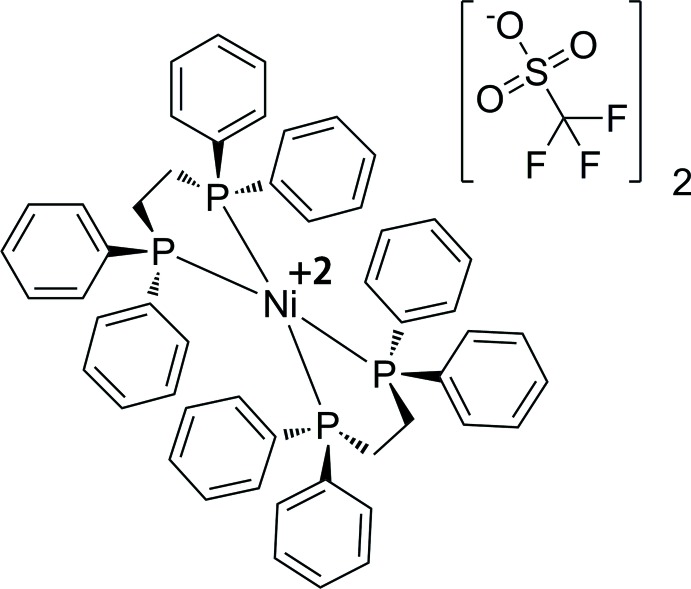


Triflates (trifluro­methane­sulfonates, CF_3_SO_4_^−^) are known as precursors of a wide range of compounds due to their lability (Lawrence, 1986 ▸). Therefore, we compare the title structure, **1**, to the structures reported with the other two counter-ions to evaluate the effect of introducing the triflate. As we describe below, the crystal structure at room temperature (see supplementary material) shows disorder of the anion that is reduced, but not completely eliminated at 100 K. In addition, the structure shows negative thermal expansion (NTE) (Liu *et al.*, 2018 ▸) based on the unit-cell volume at the two measured temperatures.

## Structural commentary   

**Figure 1:**
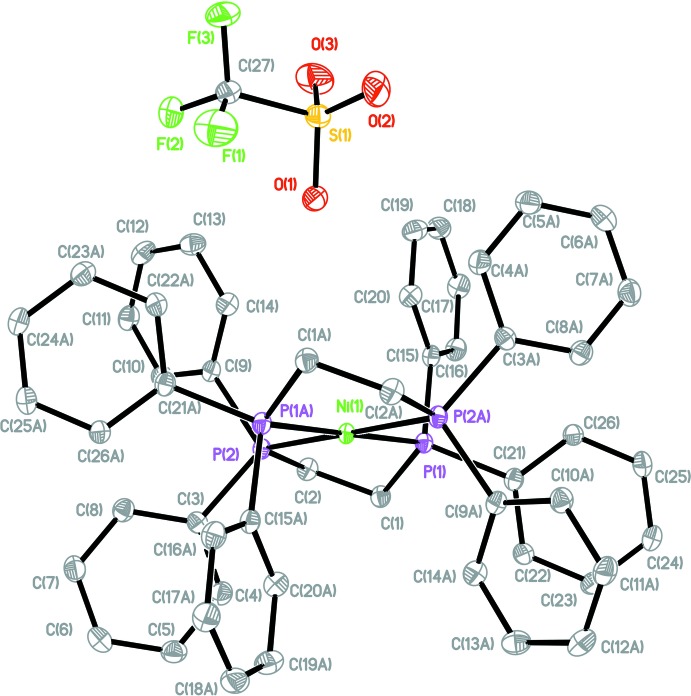
*ORTEP* rendering of **1** at 100 K with displacement ellipsoids drawn at the 50% probability level. Hydrogen atoms and the disordered parts of the anion were omitted for clarity. Atoms with the suffix A are generated by the symmetry operation (1 − *x*, 1 − *y*, 1 − *z*).

The geometry of the cation formed by Ni (site symmetry 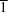) with the two dppe ligands is square planar (Fig. 1 ▸). We might expect the Ni—P distances to be the same (the ligand is symmetric); however, they are different. The corresponding distances are listed in Table 1 ▸ for the structure collected at 296 and 100 K and compared to the ones from VASCIB (Williams, 1989 ▸) and XUQYOZ (Higgs *et al.*, 2010 ▸). As this structure is formed by chelation of a simple bidentate ligand, the counter-ion has a limited effect on it, and as in the two previous structures reported, the triflate ions remain outside of the coordination sphere, being blocked from the metal center by the phenyl rings. However, there is an effect on the P—C—C—P torsion angle of the chelate ring, which is probably dependent on the size of the counter-ion (Table 1 ▸).

The bulky cation formed and the lack of strong inter­actions with the counter-ions lead to presumed dynamic disorder of the triflate ion at room temperature (296 K), which was also observed in the case of VASCIB (Williams, 1989 ▸). XUQYOZ on the other hand was acquired at a lower temperature (85 K) and no reference to any disorder was reported (Higgs *et al.*, 2010 ▸).

**Figure 2:**
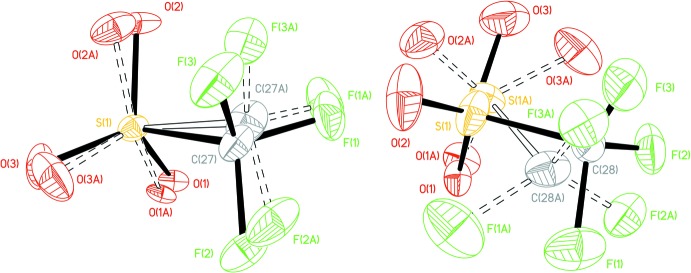
Ball and stick rendering of the tri­fluoro­methane­sulfonate ion for **1** at 100 K (left) and at 296 K (right) showing both disorder components. Open bonds indicate the minor disorder component.

For **1** at 296 K, the triflate anion is disordered over two sets of sites with 65% occupancy for the major component, which is the one with the shortest distance to the Ni atom (Fig. 2 ▸). The distance between the disordered structures is as follows, for the carbon atoms 0.744 (15) and for the S atoms 0.34 (4) Å (Fig. 2 ▸). For **1** at 100 K, the disorder is reduced although not eliminated completely (Fig. 2 ▸): the two disorder components share the S atom, while the distance between the carbon atoms is 0.354 (19) Å; the major component occupancy is similar, 67%. At 296 K there is a differentiation between the distances Ni—O from each of the parts [4.272 (8) and 4.365 (14) Å], but at 100 K the two distances are not statistically different [4.267 (8) and 4.320 (14) Å]. This could be analysed in two ways: the disorder is also static or the temperature is not low enough to eliminate completely the dynamic disorder.

Surprisingly, a negative thermal expansion was observed (Liu *et al.*, 2018 ▸). The Ni—P bond distances for **1** at 100 K (Table 1 ▸) are elongated by 1.08 and 1.20% in comparison to the values for **1** at 296 K, very close values to the volumetric expansion of the unit cell of 1.25 (12)%. With respect to the unit cell, the *a* and *b* axes are affected most in comparison with *c*, with coefficients of linear expansion (α_l_) of −29 (4) × 10^−6^, −30 (4) × 10^−6^, and −6(4) × 10 ^−6^ K^−1^ respectively. Based on two temperatures, the volumetric thermal expansion coefficient for the title compound is −63 (6) × 10 ^−6^ K^−1^.

**Figure 3:**
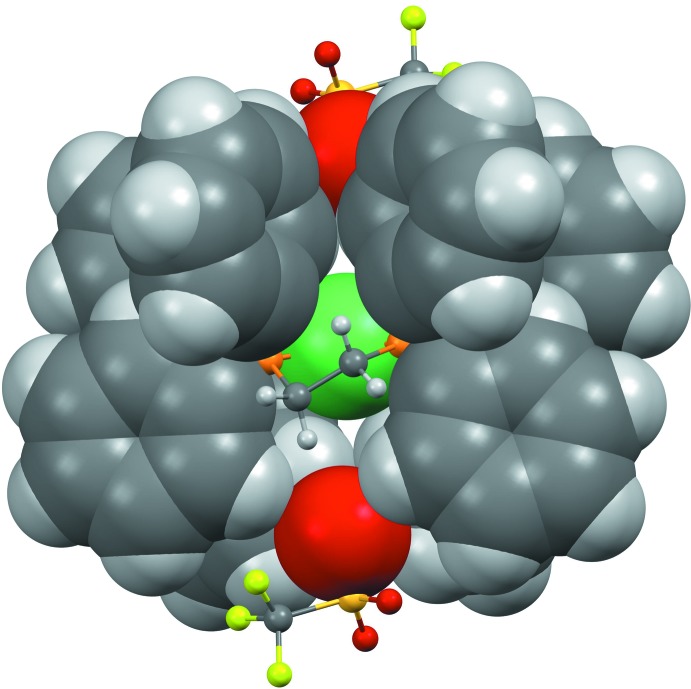
View parallel to the coordination plane of the Ni and P atoms, showing the counter-ions blocked by the phenyl rings. A space-filling rendering was used for the phenyl groups, the Ni atom and the oxygen atom pointed towards Ni. The disordered part of the anion is omitted for clarity.

Another feature of the anion–cation inter­action is that the Ni⋯O long-distance inter­action is not perpendicular to the mean plane formed by Ni and the four P atoms but tilted at an angle of 74° (Fig. 3 ▸). This tilted orientation is also present in the crystal structures of VASCIB (Williams, 1989 ▸) and XUQYOZ (Higgs *et al.*, 2010 ▸) with angles of 73 and 71°, respectively.

**Figure 4:**
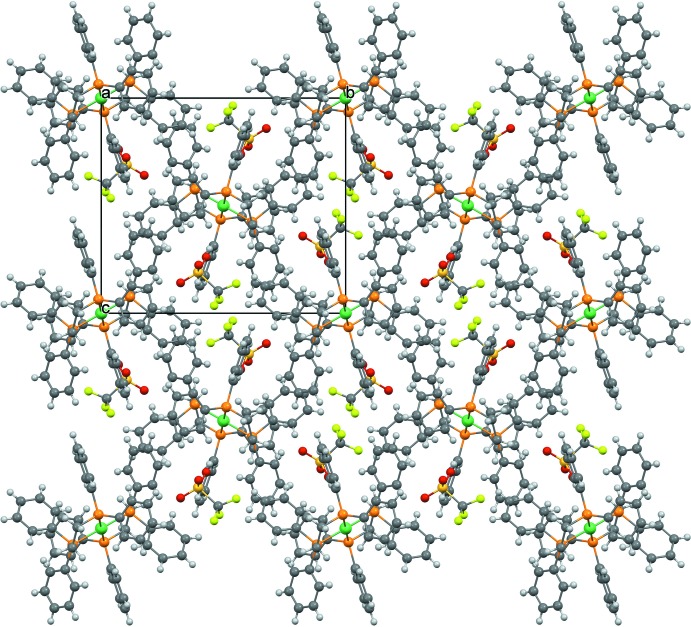
Packing view of **1** at 100 K along the *a* axis.

A packing diagram of **1** at 100 K viewed down [100] is shown in Fig. 4 ▸; there are C—H⋯*X* (*X* = O, F) interactions, but because of the disorder of the triflate ion they are not described in detail.

## Database survey   

Dppe is a very common ligand: more than 2800 structures are reported in the Cambridge Structural Database (CSD version 5.38, updated ofMay2017; Groom *et al.*, 2016 ▸), 240 of them are with nickel, and only one (LUCLOK; Uehara *et al.*, 2002 ▸) has triflate as counter-ion. In this example, as in other reports of nickel with different ligands (*e.g*. Lyubartseva *et al.*, 2013 ▸), the triflate anions are outside the coordination sphere as is the case with the title compound and with the two reports with different counter-ions: NO_3_^−^ (VASCIB; Williams, 1989 ▸) and Cl^−^ (XUQYOZ; Higgs *et al.*, 2010 ▸).

For comparison, compounds with similar structures to the title compound and the same metallic group (group 10: P^II^, Pt^II^) with bis­[1,2–bis­(di­phenyl­phosphan­yl)ethane], show almost an ideal square-planar geometry and also counter-ions outside the coordination sphere (see, for example, Engelhardt *et al.*, 1984 ▸).

With respect to the Ni—P distances, we found in the CSD that both equivalent and non-equivalent Ni—P distances occur for Ni(+2)-bis­(diphosphines), although it is hard to discern a pattern: for example, the Ni complexes formed with the 1-*para*-*X*-phenyl-3,6-triphenyl-1-aza-3,6-diphospha­cyclo­heptane ligand, *X* = Cl (IFOFOA) or Br (IFOFEQ), are isostructural compounds that crystallize in space group *P* 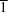 (Stewart *et al.*, 2013 ▸), but one has equivalent Ni—P bonds while the other does not.

## Synthesis and crystallization   

The title compound was prepared in two steps. First, 1,2–bis­(di­phenyl­phosphan­yl)ethane and nickel(II) chloride hexa­hydrate (molar ratio 1:2) were reacted in hot ethanol. The product obtained, di­chloro-bis­[1,2–bis­(di­phenyl­phosphan­yl)ethane]nickel(II), was then reacted with silver(I) tri­fluoro­methane­sulfonate in di­chloro­methane (molar ratio 1:2). The product of this second reaction was filtered off and purified using a Soxhlet system with di­chloro­methane in which the by product, silver(I) chloride, was insoluble (Cano, 2012 ▸).

The crystallization process was carried out by dissolution of the purified compound in the minimum volume of methanol at 323 K (≃ 2.5 mg mL^−1^). When the solution reached room temperature, it was transferred to a chamber saturated with diethyl ether. Diffusion of diethyl ether into the solution over a three-week period led to the formation of translucent intensely yellow block-like crystals at the bottom and on the walls of the vessel.

## Refinement   

Crystal data, data collection and structure refinement details are summarized in Table 2 ▸. H atoms were positioned geometrically and refined as riding with C—H = 0.95–0.99 Å and *U*_iso_(H) = 1.2*U*_eq_(C).

## Supplementary Material

Crystal structure: contains datablock(s) I. DOI: 10.1107/S2056989018014846/hb7775sup1.cif

Structure factors: contains datablock(s) I. DOI: 10.1107/S2056989018014846/hb7775Isup2.hkl

Data at 296K. DOI: 10.1107/S2056989018014846/hb7775sup3.txt

CCDC reference: 1874698

Additional supporting information:  crystallographic information; 3D view; checkCIF report

## Figures and Tables

**Table 1:** Comparison of selected geometric parameters (Å, °) for **1** at 296 and 100 K, VASCIB and XUQYOZ

Parameter	**1** at 296 K	**1** at 100 K	VASCIB	XUQYOZ
Ni—P	2.219 (2)	2.243 (1)	2.256 (3)	2.237 (1)
	2.238 (2)	2.265 (1)	2.261 (3)	2.245 (1)
P1—Ni—P2	84.7 (1)	84.9 (1)	83.2 (1)	83.6 (1)
P1—C—C—P2	43.9 (4)	42.8 (2)	30.8 (1)	39.9 (3)

**Table 2:** Experimental details

Crystal data	
Chemical formula	[Ni(C_25_H_24_P_2_)_2_](CF_3_O_3_S)_2_
*M*_r_	1153.63
Crystal system, space group	Monoclinic, *P*2_1_/*n*
Temperature (K)	100
*a*, *b*, *c* (Å)	11.0462 (4), 16.1813 (6), 14.3914 (5)
β (°)	98.143 (1)
*V* (Å^3^)	2546.41 (16)
*Z*	2
Radiation type	Mo *K*α
μ (mm^−1^)	0.66
Crystal size (mm)	0.21 × 0.19 × 0.09

Data collection	
Diffractometer	Bruker SMART APEX CCD
Absorption correction	Multi-scan (*SADABS*; Bruker, 2013 ▸)
*T*_min_, *T*_max_	0.828, 0.974
No. of measured, independent and observed [*I* > 2σ(*I*)] reflections	30965, 7253, 5928
*R*_int_	0.039
(sin θ/λ)_max_ (Å^−1^)	0.703

Refinement	
*R*[*F* ^2^ > 2σ(*F* ^2^)], *wR*(*F* ^2^), *S*	0.040, 0.099, 1.04
No. of reflections	7253
No. of parameters	395
No. of restraints	144
H-atom treatment	H-atom parameters constrained
Δρ_max_, Δρ_min_ (e Å^−3^)	0.64, −0.30

Computer programs: *APEX2* and *SAINT* (Bruker, 2013 ▸), *SHELXS97* and *SHELXTL* (Sheldrick, 2008 ▸), *SHELXL2014* (Sheldrick, 2015 ▸), *Mercury* (Macrae *et al.*, 2006 ▸), *publCIF* (Westrip, 2010 ▸) and *OLEX2* (Dolomanov *et al.*, 2009 ▸).

## supplementary crystallographic information

### Crystal data

**Table:**

[Ni(C_26_H_24_P_2_)_2_](CF_3_O_3_S)_2_	*F*(000) = 1188
*M**_r_* = 1153.63	*D*_x_ = 1.505 Mg m^−^^3^
Monoclinic, *P*2_1_/*n*	Mo *K*α radiation, λ = 0.71073 Å
*a* = 11.0462 (4) Å	Cell parameters from 8190 reflections
*b* = 16.1813 (6) Å	θ = 2.3–29.9°
*c* = 14.3914 (5) Å	µ = 0.66 mm^−^^1^
β = 98.143 (1)°	*T* = 100 K
*V* = 2546.41 (16) Å^3^	Cube, yellow
*Z* = 2	0.21 × 0.19 × 0.09 mm

### Data collection

**Table:**

Bruker SMART APEX CCD diffractometer	5928 reflections with *I* > 2σ(*I*)
Radiation source: fine-focus X-ray tube, Bruker SMART APEX CCD	*R*_int_ = 0.039
ω and φ scans	θ_max_ = 30.0°, θ_min_ = 1.9°
Absorption correction: multi-scan (SADABS; Bruker, 2013)	*h* = −15→15
*T*_min_ = 0.828, *T*_max_ = 0.974	*k* = −22→20
30965 measured reflections	*l* = −19→16
7253 independent reflections	

### Refinement

**Table:**

Refinement on *F*^2^	Primary atom site location: other
Least-squares matrix: full	Secondary atom site location: difference Fourier map
*R*[*F*^2^ > 2σ(*F*^2^)] = 0.040	Hydrogen site location: inferred from neighbouring sites
*wR*(*F*^2^) = 0.099	H-atom parameters constrained
*S* = 1.04	*w* = 1/[σ^2^(*F*_o_^2^) + (0.0417*P*)^2^ + 1.7778*P*] where *P* = (*F*_o_^2^ + 2*F*_c_^2^)/3
7253 reflections	(Δ/σ)_max_ = 0.001
395 parameters	Δρ_max_ = 0.64 e Å^−^^3^
144 restraints	Δρ_min_ = −0.30 e Å^−^^3^

### Special details

**Table:**

Geometry. All esds (except the esd in the dihedral angle between two l.s. planes) are estimated using the full covariance matrix. The cell esds are taken into account individually in the estimation of esds in distances, angles and torsion angles; correlations between esds in cell parameters are only used when they are defined by crystal symmetry. An approximate (isotropic) treatment of cell esds is used for estimating esds involving l.s. planes.

### Fractional atomic coordinates and isotropic or equivalent isotropic displacement parameters (Å^2^)

**Table:**

	*x*	*y*	*z*	*U*_iso_*/*U*_eq_	Occ. (<1)
Ni1	0.5000	0.5000	0.5000	0.01104 (8)	
P1	0.53302 (4)	0.38286 (3)	0.42411 (3)	0.01285 (10)	
P2	0.69870 (4)	0.48827 (3)	0.56393 (3)	0.01274 (10)	
S1	0.26698 (4)	0.39668 (3)	0.80990 (3)	0.02173 (11)	
F1	0.2529 (7)	0.5524 (4)	0.8625 (7)	0.0447 (13)	0.65 (2)
F2	0.4016 (7)	0.4830 (5)	0.9367 (4)	0.0379 (13)	0.65 (2)
F3	0.2175 (10)	0.4617 (6)	0.9650 (5)	0.0491 (18)	0.65 (2)
O1	0.3362 (11)	0.4294 (11)	0.7393 (10)	0.0208 (17)	0.65 (2)
O2	0.1365 (6)	0.3986 (9)	0.7762 (11)	0.039 (2)	0.65 (2)
O3	0.3121 (10)	0.3245 (4)	0.8587 (7)	0.0390 (14)	0.65 (2)
C27	0.2842 (7)	0.4783 (5)	0.8967 (5)	0.0258 (12)	0.65 (2)
F1A	0.2385 (18)	0.5530 (8)	0.8295 (13)	0.054 (3)	0.35 (2)
F2A	0.3693 (16)	0.5037 (11)	0.9377 (10)	0.052 (3)	0.35 (2)
F3A	0.1751 (13)	0.4830 (6)	0.9399 (10)	0.038 (2)	0.35 (2)
O1A	0.353 (2)	0.419 (2)	0.7470 (19)	0.018 (3)	0.35 (2)
O2A	0.1429 (12)	0.3789 (17)	0.778 (2)	0.040 (4)	0.35 (2)
O3A	0.323 (2)	0.3366 (11)	0.8793 (14)	0.052 (4)	0.35 (2)
C27A	0.2607 (15)	0.4870 (11)	0.8830 (11)	0.039 (3)	0.35 (2)
C1	0.69589 (16)	0.38126 (12)	0.41112 (13)	0.0163 (3)	
H1A	0.7191	0.3259	0.3902	0.020*	
H1B	0.7127	0.4224	0.3636	0.020*	
C2	0.76932 (16)	0.40193 (11)	0.50616 (13)	0.0163 (3)	
H2A	0.8537	0.4173	0.4974	0.020*	
H2B	0.7739	0.3525	0.5470	0.020*	
C3	0.79945 (15)	0.57547 (11)	0.55049 (12)	0.0146 (3)	
C4	0.82936 (17)	0.59377 (12)	0.46148 (13)	0.0189 (4)	
H4	0.7979	0.5603	0.4094	0.023*	
C5	0.90443 (17)	0.66028 (13)	0.44857 (14)	0.0216 (4)	
H5	0.9241	0.6724	0.3879	0.026*	
C6	0.95088 (17)	0.70922 (12)	0.52492 (14)	0.0218 (4)	
H6	1.0015	0.7552	0.5162	0.026*	
C7	0.92328 (18)	0.69083 (12)	0.61367 (14)	0.0217 (4)	
H7	0.9562	0.7238	0.6658	0.026*	
C8	0.84752 (17)	0.62430 (12)	0.62676 (13)	0.0177 (4)	
H8	0.8286	0.6122	0.6877	0.021*	
C9	0.72551 (16)	0.45953 (11)	0.68697 (12)	0.0144 (3)	
C10	0.84583 (16)	0.45136 (12)	0.73350 (13)	0.0170 (4)	
H10	0.9133	0.4647	0.7020	0.020*	
C11	0.86528 (17)	0.42372 (12)	0.82559 (13)	0.0194 (4)	
H11	0.9464	0.4189	0.8575	0.023*	
C12	0.76717 (18)	0.40297 (12)	0.87152 (13)	0.0206 (4)	
H12	0.7815	0.3842	0.9347	0.025*	
C13	0.64807 (18)	0.40952 (12)	0.82552 (14)	0.0202 (4)	
H13	0.5810	0.3948	0.8568	0.024*	
C14	0.62791 (16)	0.43798 (12)	0.73299 (13)	0.0173 (4)	
H14	0.5467	0.4426	0.7013	0.021*	
C15	0.51023 (15)	0.29773 (11)	0.50214 (12)	0.0145 (3)	
C16	0.55540 (17)	0.21858 (12)	0.48785 (13)	0.0183 (4)	
H16	0.5976	0.2081	0.4359	0.022*	
C17	0.53812 (18)	0.15561 (12)	0.55013 (14)	0.0207 (4)	
H17	0.5694	0.1020	0.5411	0.025*	
C18	0.47550 (18)	0.17043 (13)	0.62542 (14)	0.0215 (4)	
H18	0.4645	0.1270	0.6678	0.026*	
C19	0.42870 (18)	0.24842 (13)	0.63928 (14)	0.0216 (4)	
H19	0.3848	0.2583	0.6904	0.026*	
C20	0.44672 (16)	0.31192 (12)	0.57772 (13)	0.0175 (4)	
H20	0.4155	0.3655	0.5872	0.021*	
C21	0.45902 (16)	0.35263 (11)	0.30856 (12)	0.0145 (3)	
C22	0.49303 (17)	0.39130 (12)	0.22954 (13)	0.0184 (4)	
H22	0.5538	0.4333	0.2367	0.022*	
C23	0.43798 (18)	0.36837 (12)	0.14041 (13)	0.0212 (4)	
H23	0.4621	0.3941	0.0866	0.025*	
C24	0.34774 (17)	0.30791 (12)	0.12975 (13)	0.0188 (4)	
H24	0.3092	0.2932	0.0688	0.023*	
C25	0.31386 (17)	0.26894 (12)	0.20790 (13)	0.0195 (4)	
H25	0.2524	0.2274	0.2004	0.023*	
C26	0.37007 (16)	0.29075 (11)	0.29752 (13)	0.0158 (3)	
H26	0.3478	0.2635	0.3511	0.019*	

### Atomic displacement parameters (Å^2^)

**Table:**

	*U*^11^	*U*^22^	*U*^33^	*U*^12^	*U*^13^	*U*^23^
Ni1	0.01002 (14)	0.01164 (16)	0.01101 (15)	0.00041 (11)	−0.00007 (11)	−0.00175 (11)
P1	0.01274 (19)	0.0129 (2)	0.0127 (2)	−0.00012 (15)	0.00118 (16)	−0.00210 (16)
P2	0.01096 (19)	0.0140 (2)	0.0127 (2)	0.00037 (15)	−0.00016 (16)	−0.00071 (16)
S1	0.0190 (2)	0.0257 (3)	0.0215 (2)	−0.00271 (18)	0.00664 (18)	−0.00384 (19)
F1	0.059 (2)	0.0265 (18)	0.054 (3)	0.0053 (13)	0.028 (3)	−0.008 (2)
F2	0.040 (2)	0.051 (3)	0.0218 (16)	−0.0196 (18)	−0.0022 (15)	−0.0033 (15)
F3	0.063 (3)	0.058 (3)	0.035 (2)	−0.027 (3)	0.033 (2)	−0.019 (2)
O1	0.014 (3)	0.029 (4)	0.019 (2)	0.001 (3)	0.002 (2)	−0.0005 (18)
O2	0.0135 (16)	0.068 (6)	0.036 (2)	−0.0056 (18)	0.0026 (14)	−0.015 (4)
O3	0.052 (3)	0.0208 (18)	0.047 (3)	−0.0021 (16)	0.015 (2)	0.0070 (19)
C27	0.031 (2)	0.028 (2)	0.020 (2)	−0.0097 (18)	0.0108 (17)	−0.0060 (16)
F1A	0.077 (6)	0.037 (3)	0.059 (6)	0.015 (3)	0.048 (5)	0.005 (4)
F2A	0.049 (5)	0.065 (7)	0.045 (4)	−0.027 (4)	0.019 (3)	−0.026 (4)
F3A	0.049 (4)	0.038 (3)	0.036 (4)	−0.007 (3)	0.032 (3)	−0.009 (3)
O1A	0.011 (4)	0.027 (6)	0.017 (5)	0.006 (3)	0.003 (4)	−0.002 (3)
O2A	0.032 (4)	0.057 (9)	0.033 (4)	−0.014 (4)	0.014 (3)	−0.016 (5)
O3A	0.057 (6)	0.045 (6)	0.053 (7)	0.022 (5)	0.011 (5)	0.017 (5)
C27A	0.044 (5)	0.042 (5)	0.036 (5)	−0.012 (4)	0.024 (4)	−0.006 (3)
C1	0.0148 (7)	0.0181 (9)	0.0163 (8)	0.0003 (6)	0.0036 (7)	−0.0036 (7)
C2	0.0140 (7)	0.0174 (9)	0.0169 (8)	0.0037 (6)	0.0008 (6)	−0.0007 (7)
C3	0.0111 (7)	0.0153 (9)	0.0171 (8)	0.0011 (6)	0.0008 (6)	0.0005 (7)
C4	0.0170 (8)	0.0227 (10)	0.0167 (9)	−0.0017 (7)	0.0011 (7)	−0.0014 (7)
C5	0.0195 (8)	0.0256 (10)	0.0202 (9)	−0.0013 (7)	0.0046 (7)	0.0028 (8)
C6	0.0194 (8)	0.0186 (10)	0.0275 (10)	−0.0031 (7)	0.0036 (8)	0.0003 (8)
C7	0.0230 (9)	0.0195 (10)	0.0219 (9)	−0.0034 (7)	0.0005 (8)	−0.0029 (7)
C8	0.0194 (8)	0.0168 (9)	0.0168 (8)	0.0011 (7)	0.0017 (7)	−0.0011 (7)
C9	0.0158 (7)	0.0136 (8)	0.0131 (8)	0.0016 (6)	0.0002 (6)	−0.0011 (6)
C10	0.0150 (8)	0.0180 (9)	0.0172 (8)	0.0009 (6)	−0.0003 (7)	0.0007 (7)
C11	0.0201 (8)	0.0186 (9)	0.0177 (9)	0.0035 (7)	−0.0033 (7)	0.0006 (7)
C12	0.0275 (9)	0.0194 (9)	0.0145 (8)	0.0057 (7)	0.0018 (7)	0.0006 (7)
C13	0.0217 (9)	0.0204 (10)	0.0199 (9)	0.0030 (7)	0.0074 (7)	0.0012 (7)
C14	0.0162 (8)	0.0184 (9)	0.0173 (8)	0.0023 (7)	0.0017 (7)	−0.0004 (7)
C15	0.0141 (7)	0.0147 (8)	0.0140 (8)	0.0003 (6)	−0.0010 (6)	−0.0011 (6)
C16	0.0188 (8)	0.0182 (9)	0.0178 (8)	0.0022 (7)	0.0027 (7)	−0.0032 (7)
C17	0.0247 (9)	0.0152 (9)	0.0211 (9)	0.0034 (7)	0.0001 (8)	0.0003 (7)
C18	0.0222 (9)	0.0212 (10)	0.0204 (9)	−0.0007 (7)	0.0006 (7)	0.0052 (7)
C19	0.0215 (9)	0.0268 (11)	0.0174 (9)	0.0013 (8)	0.0053 (7)	0.0026 (7)
C20	0.0177 (8)	0.0179 (9)	0.0170 (8)	0.0024 (7)	0.0025 (7)	−0.0002 (7)
C21	0.0156 (7)	0.0129 (8)	0.0148 (8)	0.0018 (6)	0.0011 (6)	−0.0026 (6)
C22	0.0223 (8)	0.0162 (9)	0.0168 (9)	−0.0044 (7)	0.0024 (7)	−0.0013 (7)
C23	0.0264 (9)	0.0218 (10)	0.0155 (9)	−0.0016 (8)	0.0029 (7)	0.0003 (7)
C24	0.0199 (8)	0.0200 (9)	0.0153 (8)	0.0009 (7)	−0.0012 (7)	−0.0035 (7)
C25	0.0192 (8)	0.0176 (9)	0.0205 (9)	−0.0026 (7)	−0.0013 (7)	−0.0036 (7)
C26	0.0180 (8)	0.0143 (9)	0.0152 (8)	−0.0014 (6)	0.0026 (7)	−0.0015 (6)

### Geometric parameters (Å, º)

**Table:**

Ni1—P1	2.2431 (5)	C3—C8	1.395 (2)
Ni1—P1^i^	2.2431 (5)	C3—C4	1.399 (3)
Ni1—P2	2.2646 (4)	C4—C5	1.387 (3)
Ni1—P2^i^	2.2646 (4)	C5—C6	1.392 (3)
P1—C21	1.8132 (18)	C6—C7	1.387 (3)
P1—C15	1.8172 (19)	C7—C8	1.393 (3)
P1—C1	1.8350 (18)	C9—C14	1.387 (3)
P2—C9	1.8141 (18)	C9—C10	1.407 (2)
P2—C3	1.8243 (19)	C10—C11	1.386 (3)
P2—C2	1.8533 (18)	C11—C12	1.388 (3)
S1—O2A	1.413 (12)	C12—C13	1.390 (3)
S1—O3	1.416 (6)	C13—C14	1.397 (3)
S1—O1A	1.442 (12)	C15—C20	1.394 (3)
S1—O2	1.454 (6)	C15—C16	1.400 (3)
S1—O1	1.455 (7)	C16—C17	1.388 (3)
S1—O3A	1.467 (11)	C17—C18	1.386 (3)
S1—C27A	1.807 (17)	C18—C19	1.389 (3)
S1—C27	1.809 (7)	C19—C20	1.389 (3)
F1—C27	1.323 (6)	C21—C22	1.395 (3)
F2—C27	1.345 (6)	C21—C26	1.396 (2)
F3—C27	1.337 (6)	C22—C23	1.390 (3)
F1A—C27A	1.319 (11)	C23—C24	1.390 (3)
F2A—C27A	1.366 (11)	C24—C25	1.386 (3)
F3A—C27A	1.337 (11)	C25—C26	1.395 (2)
C1—C2	1.525 (2)		
			
P1—Ni1—P1^i^	179.999 (19)	F3A—C27A—F2A	107.2 (11)
P1—Ni1—P2	84.943 (16)	F1A—C27A—S1	109.5 (10)
P1^i^—Ni1—P2	95.056 (16)	F3A—C27A—S1	114.0 (11)
P1—Ni1—P2^i^	95.057 (16)	F2A—C27A—S1	113.2 (10)
P1^i^—Ni1—P2^i^	84.945 (16)	C2—C1—P1	108.06 (12)
P2—Ni1—P2^i^	180.0	C1—C2—P2	111.31 (12)
C21—P1—C15	106.16 (8)	C8—C3—C4	119.18 (17)
C21—P1—C1	102.94 (8)	C8—C3—P2	121.64 (14)
C15—P1—C1	106.01 (8)	C4—C3—P2	119.18 (14)
C21—P1—Ni1	126.24 (6)	C5—C4—C3	120.67 (18)
C15—P1—Ni1	107.06 (6)	C4—C5—C6	119.74 (19)
C1—P1—Ni1	106.91 (6)	C7—C6—C5	120.03 (18)
C9—P2—C3	106.51 (8)	C6—C7—C8	120.34 (18)
C9—P2—C2	102.93 (8)	C7—C8—C3	120.03 (18)
C3—P2—C2	103.51 (8)	C14—C9—C10	119.65 (16)
C9—P2—Ni1	115.65 (6)	C14—C9—P2	120.00 (13)
C3—P2—Ni1	117.57 (6)	C10—C9—P2	120.08 (14)
C2—P2—Ni1	109.04 (6)	C11—C10—C9	119.56 (17)
O2A—S1—O1A	122.4 (18)	C10—C11—C12	120.49 (17)
O3—S1—O2	116.5 (7)	C11—C12—C13	120.31 (17)
O3—S1—O1	117.6 (7)	C12—C13—C14	119.43 (18)
O2—S1—O1	110.7 (9)	C9—C14—C13	120.55 (16)
O2A—S1—O3A	112.6 (15)	C20—C15—C16	119.63 (17)
O1A—S1—O3A	109.8 (13)	C20—C15—P1	119.09 (14)
O2A—S1—C27A	103.9 (12)	C16—C15—P1	121.28 (14)
O1A—S1—C27A	104.4 (16)	C17—C16—C15	119.48 (18)
O3A—S1—C27A	100.8 (8)	C18—C17—C16	120.48 (18)
O3—S1—C27	105.3 (4)	C17—C18—C19	120.43 (18)
O2—S1—C27	102.5 (6)	C18—C19—C20	119.38 (18)
O1—S1—C27	101.7 (9)	C19—C20—C15	120.59 (18)
F1—C27—F3	108.3 (6)	C22—C21—C26	119.69 (16)
F1—C27—F2	107.0 (6)	C22—C21—P1	119.21 (14)
F3—C27—F2	107.1 (5)	C26—C21—P1	121.10 (14)
F1—C27—S1	114.1 (5)	C23—C22—C21	119.96 (17)
F3—C27—S1	110.3 (5)	C24—C23—C22	120.20 (18)
F2—C27—S1	109.7 (5)	C25—C24—C23	120.17 (17)
F1A—C27A—F3A	107.9 (11)	C24—C25—C26	119.94 (17)
F1A—C27A—F2A	104.5 (13)	C25—C26—C21	120.03 (17)
			
O3—S1—C27—F1	176.3 (7)	Ni1—P2—C9—C14	6.68 (17)
O2—S1—C27—F1	−61.4 (9)	C3—P2—C9—C10	−46.75 (17)
O1—S1—C27—F1	53.1 (7)	C2—P2—C9—C10	61.80 (16)
O3—S1—C27—F3	−61.6 (7)	Ni1—P2—C9—C10	−179.41 (13)
O2—S1—C27—F3	60.7 (9)	C14—C9—C10—C11	−1.5 (3)
O1—S1—C27—F3	175.2 (6)	P2—C9—C10—C11	−175.45 (15)
O3—S1—C27—F2	56.2 (7)	C9—C10—C11—C12	0.9 (3)
O2—S1—C27—F2	178.5 (8)	C10—C11—C12—C13	0.1 (3)
O1—S1—C27—F2	−67.0 (7)	C11—C12—C13—C14	−0.7 (3)
O2A—S1—C27A—F1A	−77.5 (16)	C10—C9—C14—C13	1.0 (3)
O1A—S1—C27A—F1A	51.8 (14)	P2—C9—C14—C13	174.95 (15)
O3A—S1—C27A—F1A	165.7 (13)	C12—C13—C14—C9	0.1 (3)
O2A—S1—C27A—F3A	43.5 (16)	C21—P1—C15—C20	−119.49 (14)
O1A—S1—C27A—F3A	172.8 (13)	C1—P1—C15—C20	131.50 (14)
O3A—S1—C27A—F3A	−73.3 (13)	Ni1—P1—C15—C20	17.63 (15)
O2A—S1—C27A—F2A	166.3 (15)	C21—P1—C15—C16	60.66 (16)
O1A—S1—C27A—F2A	−64.4 (14)	C1—P1—C15—C16	−48.34 (16)
O3A—S1—C27A—F2A	49.5 (14)	Ni1—P1—C15—C16	−162.22 (13)
C21—P1—C1—C2	−177.35 (12)	C20—C15—C16—C17	−1.1 (3)
C15—P1—C1—C2	−66.06 (14)	P1—C15—C16—C17	178.74 (14)
Ni1—P1—C1—C2	47.91 (13)	C15—C16—C17—C18	0.7 (3)
P1—C1—C2—P2	−42.78 (16)	C16—C17—C18—C19	0.3 (3)
C9—P2—C2—C1	143.54 (13)	C17—C18—C19—C20	−0.9 (3)
C3—P2—C2—C1	−105.67 (14)	C18—C19—C20—C15	0.5 (3)
Ni1—P2—C2—C1	20.24 (14)	C16—C15—C20—C19	0.5 (3)
C9—P2—C3—C8	−20.77 (17)	P1—C15—C20—C19	−179.36 (14)
C2—P2—C3—C8	−128.91 (15)	C15—P1—C21—C22	−159.38 (15)
Ni1—P2—C3—C8	110.83 (14)	C1—P1—C21—C22	−48.20 (17)
C9—P2—C3—C4	159.16 (14)	Ni1—P1—C21—C22	74.38 (16)
C2—P2—C3—C4	51.03 (16)	C15—P1—C21—C26	19.77 (17)
Ni1—P2—C3—C4	−69.23 (15)	C1—P1—C21—C26	130.94 (15)
C8—C3—C4—C5	−0.9 (3)	Ni1—P1—C21—C26	−106.47 (14)
P2—C3—C4—C5	179.16 (15)	C26—C21—C22—C23	0.3 (3)
C3—C4—C5—C6	0.2 (3)	P1—C21—C22—C23	179.44 (15)
C4—C5—C6—C7	0.8 (3)	C21—C22—C23—C24	1.0 (3)
C5—C6—C7—C8	−1.1 (3)	C22—C23—C24—C25	−1.3 (3)
C6—C7—C8—C3	0.3 (3)	C23—C24—C25—C26	0.3 (3)
C4—C3—C8—C7	0.6 (3)	C24—C25—C26—C21	1.0 (3)
P2—C3—C8—C7	−179.43 (14)	C22—C21—C26—C25	−1.3 (3)
C3—P2—C9—C14	139.35 (15)	P1—C21—C26—C25	179.59 (14)
C2—P2—C9—C14	−112.11 (16)		

Symmetry code: (i) −*x*+1, −*y*+1, −*z*+1.
